# Treating the invisible: Gaps and opportunities for enhanced TB control along the Thailand-Myanmar border

**DOI:** 10.1186/s12913-016-1954-9

**Published:** 2017-01-13

**Authors:** Naomi Tschirhart, Sein Sein Thi, Lei Lei Swe, Francois Nosten, Angel M. Foster

**Affiliations:** 1 Faculty of Health Sciences, University of Ottawa, 1 Stewart Street, Ottawa, K1N 6N5 ON Canada; 2 Shoklo Malaria Research Unit, Mahidol-Oxford Tropical Medicine Research Unit, Faculty of Tropical Medicine, Mahidol University, PO Box 46, Mae Sot, Tak 63110 Thailand; 3 Centre for Tropical Medicine and Global Health, Nuffield Department of Clinical Medicine, University of Oxford, Churchill Hospital, Oxford, UK

**Keywords:** Tuberculosis, Migrants, Surveillance, Treatment, MDR-TB, TB/HIV

## Abstract

**Background:**

In Thailand’s northwestern Tak province, contextual conditions along the border with Myanmar pose difficulties for TB control among migrant populations. Incomplete surveillance data, migrant patient mobility, and loss to follow-up make it difficult to estimate the TB burden and implement effective TB control measures. This multi-methods study examined tuberculosis, tuberculosis and human immunodeficiency virus co-infection, and multidrug-resistant tuberculosis treatment accessibility for migrants and refugees in Tak province, health system response, and public health surveillance.

**Methods:**

In this study we conducted 13 interviews with key informants working in public health or TB treatment provision to elicit information on TB treatment availability and TB surveillance practices. In addition we organized 15 focus group discussions with refugee and migrant TB, TB/HIV, and MDR-TB patients and non-patients to discuss treatment access. We analyzed the data using thematic analysis and created treatment availability maps with Google maps.

**Results:**

The study identified surveillance, treatment, and funding gaps. Migrant TB cases are underreported in the provincial statistics due to jurisdictional interpretations and resource barriers. Our results suggest that TB/HIV and MDR-TB treatment options are limited for migrants and a heavy reliance on donor funding may lead to potential funding gaps for migrant TB services. We identified several opportunities that positively contribute to TB control in Tak province: improved diagnostics, comprehensive care, and collaboration through data sharing, planning, and patient referrals. The various organizations providing TB treatment to migrant and refugee populations along the border and the Tak Provincial Public Health Office are highly collaborative which offers a strong foundation for future TB control initiatives.

**Conclusions:**

Our findings suggest the need to enhance the surveillance system to include all migrant TB patients who seek treatment in Tak province and support efforts by stakeholders on both sides of the border to continue to share data and engage in collaborative planning on TB, TB/HIV, and MDR-TB treatment provision for migrant populations.

## Background

Tuberculosis (TB) control across international borders has been identified as a challenge in multiple geographic contexts [1–4]. Migrants with TB may experience challenges accessing early TB diagnosis, lack of continuity of care, and difficulties accessing healthcare in the new country [1]. From a surveillance perspective, data collection remains a barrier to effective TB control as public health departments struggle to document the number HIV treatment for this population. Since cases among mobile patients in their jurisdiction as well as to share and collaborate with their counterparts on the other side of the national border. Surveillance, defined as the “continual analysis, interpretation, and feedback of systematically collected data” can be challenging in border regions [5].

In South-East Asia increased mobility across international borders poses difficulties for effective infectious disease control [6]. Thailand is host to 2.7 million migrant workers from the neighboring countries of Cambodia, Lao Peoples’ Democratic Republic, and Myanmar as well as 127,000 persons living in shelters near the Thailand-Myanmar border [7]. It is expected that migration into Thailand will increase with the economic integration of the Association of Southeast Asian Nations (ASEAN) and the ASEAN Economic Community (AEC) established in 2015. While the AEC has introduced policies to encourage labour mobility for skilled workers, it is anticipated that migration of low skilled workers between ASEAN countries will also increase due to freer trade and associated investment and economic development [8]. Increasing numbers of migrants have implications for TB control in Thailand as well as the potential augmented need for health care services in the border regions [7, 9]. Migrants may be vulnerable to illness due to poor living and working conditions and challenges accessing healthcare [7].

Multi-drug resistant tuberculosis (MDR-TB) has been identified as an emerging infectious disease in South-East Asia, yet because of weaknesses and differences in the national surveillance systems in the region it is difficult to estimate and compare the burden for this and other emerging infectious diseases [6]. Under reporting of emerging infectious disease has the potential to hamper prevention and an effective public health response [6]. In Thailand lack of complete surveillance data, inclusive of migrant cases, has been identified as a hindrance to health policy and health services development [7]. A 2007 demonstration project collected TB data from all government and non-government care providers in Tak province including non-Thai patient data, however the authors indicated that data are not uniformly reported to the National TB Programme [10]. Along the Thailand-Myanmar border limited coordination among stakeholders, unstructured information sharing, loss to follow-up, and limited resources have been identified as challenges to cross-border TB control as well as for control within the respective Thailand and Myanmar border regions [11].

Tak province, Thailand is situated in the country’s northwestern region and shares a 500 km border with Kayin state, Myanmar. In Tak province public health surveillance is overseen by the Tak Provincial Public Health Office (Tak PHO) with guidance on TB surveillance from Thailand’s National Tuberculosis Programme. Historical surveillance data from Tak province from 2006 to 2011 indicate that most of the TB cases occurred in Mae Sot and the majority of these were among non-Thais [12]. Mae Sot is the city in Tak province which is closest to the Thailand-Myanmar border. A 2007 study also found that the majority HIV treatment for this population. Since cases (65%) were among non-Thais in Tak province and estimated the prevalence to be 109 per 100,000 for Thai citizens, 340 per 100,000 for Myanmar refugees, and 150 per 100,000 for Myanmar migrants [10]. On the Myanmar side of the border, directly across the border from Tak province, in Myawadee township the case notification rate of new smear positive TB cases was 178/100,000 in 2012 [9].

In Tak province five Thai government hospitals and three organizations, Première Urgence-Aide Médicale Internationale (PU-AMI), the Shoklo Malaria Research Unit (SMRU), and the International Organization for Migration (IOM), treat migrants and refugees who have TB. Access to healthcare is closely related to legal status and some organizations provide TB treatment specifically for migrants and/or refugees who are not eligible to receive low cost care from the Thai government system. All of the organizations that provide TB treatment to migrants and refugees in Tak province belong to the Tak Tuberculosis Initiative (TTBI) which provides a forum for organizations to share data and develop shared strategies for TB control in the border region. Beyond the TTBI there is additional evidence of cross-border collaboration as official transfer forms have been developed for cross border referral. While key informants in a 2014 study indicated that the border referral system needs further improvements to be of practical use, the study documents that preliminary border referral discussions have begun [11].

Our overall research project aimed to examine TB treatment accessibility for migrants and refugees in Tak province, provincial TB surveillance, and health system response to treatment barriers. We have published two manuscripts from this project. The first documents pathways to treatment and travel with TB [13]. The second focuses on access to TB treatment for migrants and refugees [14]. In this article we discuss the gaps and opportunities for improved TB control in the border region of Tak province and examine TB control from a wider lens through emphasis on surveillance. We use the term “TB control” throughout to indicate TB care and prevention efforts and acknowledge that TB control is not solely dependent on public health specialists but also relies to the resources HIV treatment for this population. Since patients and their families [5].

## Methods

Our team conducted primary data collection in Tak province, Thailand from August-October, 2014 with one additional follow up interview in December 2015. To collect information from different perspectives, we organized interviews with key informants (KIs) working in public health or TB treatment provision, as well as focus group discussions (FGDs) with TB patients and non-patients, and a survey with community health volunteers. As our participants had varying levels of literacy and legal statuses within Thailand we opted to collect verbal consent from all participants. We read the consent form to participants and documented their verbal consent. Collecting verbal consent allowed us to further safeguard participants’ identities, as we could avoid carrying around documentation with the names of informants. We have detailed our research methods elsewhere [13, 14]. This paper reports on the findings from the interviews and FGDs that are relevant to TB control.

### Data collection

Thirteen individuals who were providing TB treatment, supportive care, or were working in a public health capacity participated in the KI interviews. We compiled a list of organizations working on TB and public health in the border region and recruited KI participants at these organizations through email and phone communications. To be eligible to participate individuals had to be working in an organization that contributes to infectious disease surveillance or that provides TB treatment or supportive care to migrants and/or refugees in Tak province. We interviewed each KI their place of work and the majority of interviews took place in the Mae Sot border district of Tak province where many organizations working along the border have administrative offices. NT led the interviews with assistance from a Thai interpreter when needed. The interviews focused on TB, TB/HIV, and MDR-TB treatment and surveillance based on the participant’s professional experience. Specifically, we asked clinic staff and administrators about their TB care programs, patient barriers to care, and responsive actions aimed at improving access to care. We asked participants who collected and analyzed TB records about data collection practices, associated challenges, and changes that had been implemented in the previous 2 years.

We held 15 FGDs with migrants and refugees who were living or seeking healthcare in Tak province. The groups were disaggregated by health status, gender, and migrant or refugee identification. For this project we define migrants as individuals who have resided in a foreign country for more than 1 month or who have crossed a national border to access essential services. We use the term undocumented migrant to refer to individuals that do not have the necessary documentation to travel legally. In recruiting refugees, we included individuals who had received refugee status in the refugee camp. We held 11 FGDs with TB, TB/HIV, and MDR-TB patients (*n* = 61) and 4 FGD with non-patients (*n* = 31). Table 1 provides descriptive information on the composition of the FGDs.

**Table 1:** Description of Focus Group Discussions

FGD	Location	Description	Number of participants	Participant status
1	Mae La TB village	Men with TB	6	Refugees and Migrants
2	Mae La TB village	A man and woman with active TB	2	Refugees
3	Mae La TB village	Women with TB	5	Refugees and migrants
4	Mae La TB village	Men who do not have TB	7	Refugees
5	Mae La TB village	Women who do not have TB	8	Refugees
6	SMRU TB village	Women with TB	5	Migrants
7	SMRU TB village	Men with TB	7	Migrants
8	SMRU TB village	Women with TB/HIV	7	Migrants
9	SMRU TB village	Men with TB/HIV	8	Migrants
10	SMRU TB village	Women with MDR-TB	6	Refugees and migrants
11	SMRU TB village	Men with MDR-TB	7	Refugee and migrants
12	Mae Sot Hospital	Women with TB	3	Migrants
13	Mae Sot Hospital	Women and Men with TB	5	Migrants
14	Community health post	Men who do not have TB	8	Migrants
15	Community health post	Women who do not have TB	8	Migrants

We recruited patients from TB clinics operated by SMRU, the Mae Sot Hospital, and PU-AMI. TB doctors and clinic staff told eligible individuals about the study and conveyed that participation was voluntary and that their decision to participate would not affect their care. Doctors and clinic staff informed interested patients of the location and time of the FGD. To be eligible to participate, patients needed to have a confirmed case HIV treatment for this population. Since, MDR-TB or TB/HIV. We recruited non-patients with the assistance of PU-AMI in the refugee camp and World Vision Thailand (WVT) in migrant residential communities in Mae Sot. Program staff informed eligible participants of the voluntary study as well as the time and place that the FGD would occur.

FGDs took place at the SMRU TB village, the PU-AMI TB village, the Mae Sot Hospital, and at two World Vision migrant health posts. In each location we conducted the FGDs in a separate area from where treatment was being given. After participants consented to participate, NT conducted the FGD with assistance from two interpreters who translated from English to Burmese and Karen languages. FGDs explored participants’ actual or perceived ability to access TB treatment as well as the related barriers and enabling factors. Participants received 150 baht (approximately 4 USD) as a reimbursement for travel expenses.

### Data analysis

We transcribed FGDs and KI interviews verbatim and conducted thematic analysis by coding the data in NVivo for both deductive and emergent themes. We analyzed the themes of treatment access, surveillance, and health system characteristics separately and synthesized the findings in the final analytic phase to identify the gaps and opportunities for TB control. In addition to our thematic analysis we used the data to identify where migrants and refugees could receive free TB treatment and created maps of treatment availability using Google maps software. In June 2015 NT returned to Mae Sot, Thailand to present the maps and our preliminary findings to stakeholders as a member checking exercise to gain further input and improve the quality our findings [15]. NT conducted the analysis and received support on interpreting the findings from ST, LS, FN and AF.

## Results

The results from both components of the project suggest that there are both gaps and opportunities related to TB control. We describe the results around three gaps, surveillance, treatment, and funding and three opportunities, diagnostics, comprehensive care, and inter-organizational collaboration. Throughout this section we use illustrative quotes to showcase themes and ideas. We have redacted or masked all personally identifying information and use pseudonyms throughout the paper. We have chosen fictitious names that reflect the participant’s gender and ethnicity.

### Surveillance gaps

Based on the data from our KI interviews we identified a variety of data collection and reporting practices. The Tak PHO collects data on the number of Thai and non-Thai TB cases under its mandate from the National TB Program. The Tak PHO data is a compilation of information sent from the provincial Thai government hospitals that is integrated into a web based database and then forwarded to the National TB Program. SMRU and PU-AMI collect rigorous data from their TB patients and submit reports to Tak PHO and their funders. IOM also collects data and shares this with the refugee’s resettlement country. In Thailand refugees are under the jurisdiction of the Ministry of the Interior and subsequently IOM reports on its activities to this ministry as well.

We identified some gaps in the surveillance system as TB cases treated by non-governmental providers outside of the refugee camps are not included in the provincial statistics. SMRU predominantly treats migrants but also provides MDR-TB treatment to refugees. A key informant working in a local public health capacity explained that as SMRU treats cross-border patients, these numbers are not included in the provincial numbers. While PU-AMI treats refugees with TB and submits their reports to Tak PHO, it is not clear if the numbers are accounted for in the provincial statistics.

Key informants identified jurisdictional and resource barriers to integrating data on TB cases treated by non-governmental organizations (NGOs) into the provincial surveillance system. From a jurisdictional perspective, in Tak province cross-border populations that come across the border to access healthcare are not included in the general surveillance statistics. Research participants had divergent opinions about whether migrants who live in Myanmar but obtain healthcare in Tak province, Thailand should be included in the provincial and national TB statistics. Local public health officials also noted resource challenges related to integrating the TB data from different organizations into the surveillance system. A previous Tak PHO and Thailand-United States of America cooperation funded pilot project had collected TB records from all government hospitals and NGO health clinics in Tak province, however this collaboration ended and NGO data was not subsequently included in the provincial statistics.

We observed that organizations have different protocols for active screening, which could make it difficult to meaningfully combine data for surveillance purposes. For example IOM screens all refugees who are part of the resettlement scheme for TB; adults get a chest x-ray and children under the age of 15 first receive a tuberculin skin test. As IOM screens everyone it is anticipated that their prevalence rates are higher than the other organizations that only routinely screen high-risk groups. In the refugee camp PU-AMI uses questionnaires, sputum tests, and chest x-rays to screen contact cases, new arrivals, HIV patients, healthcare workers, and patients who have diabetes or hypertension. SMRU screens healthcare workers and provides contact case tracing and screening for family members and contacts of TB patients who are in Thailand. SMRU also does active TB screening among HIV infected persons identified through a mother to child prevention program. Patients, their partners, and their children are screened for TB using a clinical questionnaire and chest x-ray for adults and a tuberculin skin test, chest x-ray and clinical screening for children less than 5 years old. Mae Sot Hospital (MSH) and the Tak PHO also participate in active screening programs. MSH conducts screening in migrant communities, prisons, and with Thai patients who are living with chronic disease. Tak PHO actively screens contact cases and patients living with chronic disease. Key informants working in organizations that provided TB treatment indicated that TB patients received HIV counseling and were offered HIV testing. These organizations also collected data on the number of HIV co-infections.

### Treatment gaps

**Fig. 1:**
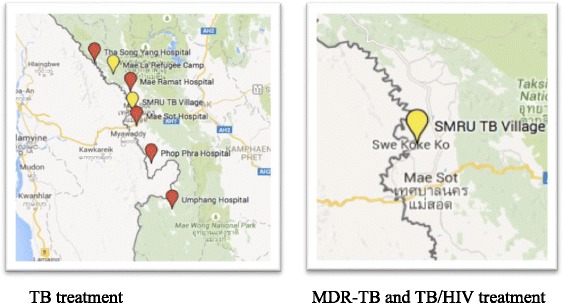
Location of treatment availability for un-documented migrants: TB vs MDR-TB and TB/HIV. NGO clinics are marked in *yellow* and Thai government hospitals are marked in *red*. Image similar but not identical to original created using Google Maps and therefore used for illustrative purposes only

Treatment plays an important role in TB control. Our results suggest that in Tak province access to TB treatment is related to legal status [14]. Migrants who have enrolled in the Thai Compulsory Migrant Health Insurance Scheme are eligible to access low cost TB treatment at the Thai government hospital. From 2011 to 2014 undocumented migrants were also able to access treatment at the Thai hospital as their treatment was funded by a grant from the European Union. Undocumented migrants could also receive TB treatment from SMRU and PU-AMI. The mapping of treatment availability by legal status and TB subtype showed that TB treatment options for migrants become more limited as the care they need becomes more complex. Figure 1 shows that there are multiple locations where undocumented migrants can get TB care in the border region of Tak province, while there is only one provider for MDRTB and TB/HIV. SMRU is the sole provider of MDR-TB treatment to undocumented migrants. SMRU also provides MDR-TB treatment to refugees who are referred from the refugee camps by PU-AMI. SMRU has two TB treatment centres along the border, one on the Thailand side and one in KoKo, Myanmar.

While the focus of our research was on TB, we discovered that HIV treatment for migrants in Tak province is also limited and found that SMRU is the primary provider of TB/HIV treatment for this population. Since 2014, PU-AMI has also provided TB/HIV treatment to patients regardless of status as refugees or migrants. Our team collected data from two of five district hospitals in Tak province. At the time of the study, one Thai government hospital in the border region, did not provide HIV treatment for migrants who had TB but rather only treated them for TB. A key informant from the other government hospital indicated that access to HIV treatment for migrants at their hospital was limited for two reasons. First, there is a restricted amount of funding allocated for HIV treatment for migrant patients and secondly migrants’ mobility often render them ineligible for treatment as they may miss follow up appointments which could contribute to drug resistance. A physician working primarily with refugee populations expressed the challenge of mitigating drug resistance while providing HIV treatment for patients, “Because it’s a long term treatment. If they are from outside, from across the border it’s not easy to follow up. It can become resistant if they do not take the treatment regularly.”

In some aspects having TB provides patients with preferential access to HIV treatment. A key informant working at a migrant healthcare clinic explained, “A patient with TB and HIV is luckier because SMRU after they treat the TB they have to treat for the TB and HIV too. So we can say lucky.” SMRU also has a prevention of mother-to-child transmission program under which eligible women and their partners receive HIV treatment. Migrants who have HIV can also seek care at the Mae Tao Clinic, a locally run NGO, which provides HIV treatment to a limited number of patients.

### Funding gaps

We identified potential funding gaps due to a lack of sustainable financing for TB treatment. For migrants with TB in Tak province, supportive care and medical treatment is closely linked to donor funding. In 2014 when we collected these data, WVT was completing a supportive care project that provided food, transportation, and daily medication to TB patients. WVT was finalizing the project and waiting for funds for its next activities. Similarly SMRU had received funding that was finishing at the end of 2014 and 2015. In addition the funding received was for a limited quota of patients. A doctor running the TB program explained the dilemma,

So we are facing a problem because our contract is through and they have additional diagnosed patients. Even for us if we diagnose a new case. I don’t know how to offer the service to this person. To refer them back to Myanmar?

The key informant also indicated that they had located additional cases by using GeneXpert MTB/RIF assay, a test that can identify TB and resistance to rifampicin, one of the drugs that is commonly used for TB treatment [16]. The GeneXpert test can provide results in less than 2 hours [16]. The doctor expressed that increased case findings from the GeneXpert test further compound funding shortages. Patients also indicated concern about the funding shortage. Wiya Htoo a male MDR-TB patient who had been working as a medic at a migrant health clinic when he fell ill expressed, “I worry about my family and other people, it’s hard to tell if they will have this disease. If they get it what should we do if there is no MDR-TB treatment available?”

One of the challenges associated with obtaining donor funding for Tak province noted by one of the KIs, is that as Thailand is now considered as an upper-middle income country it may become increasingly difficult to apply for international donor funds. As treatment for migrants and refugees is predominantly provided by local and international NGOs along the Thailand-Myanmar border, there is also the potential for a gap in treatment provision when an organization ceases to operate in the border area. According to a key informant this happened when Médecins Sans Frontières (MSF) ceased its operations in Thailand in 2011.

### Opportunity: Improved diagnostics

When asked if there had been any changes in methods used for TB screening in the previous 2 years, KI participants described an increase in the use of the GeneXpert test. One informant explained that GeneXpert was initially only used in their organization for suspected drug resistant cases but subsequently all suspected TB patients receive GeneXpert testing. At the time of our study, PU-AMI, SMRU, and IOM provided GeneXpert testing to all of their TB patients. Participants explained that GeneXpert creates opportunities for enhanced TB control by assisting in the identification of individuals with drug resistant TB. A doctor running a TB program noted the benefits of GeneXpert, “Increased MDR-TB case findings in migrants and refugees in this area may be due to the use of GeneXpert test. We see cases more quickly and more and more”. However, as described above, key informants articulated that the opportunities brought about by new case findings pose a challenge for clinicians as there is limited availability of MDR-TB treatment for migrants and refugees in Tak province.

### Opportunity: Comprehensive programs with good treatment adherence

While migration and patient disappearance or relocation during TB treatment poses challenges for adherence, during our analysis we found that organizations in Tak province have developed residential treatment programs and supportive care to decrease default rates among migrants. A default rate of 12.7% for non-Thai TB patients in Tak province was reported in 2012 [9]. However, SMRU, which has a residential program, had a lower default rate 5% in 2012 and 4% in 2013. Both SMRU and PU-AMI have TB villages where patients can stay during their treatment. Patients indicated that the comprehensive care, inclusive of medical treatment and supportive services, received at the TB village helped them adhere to treatment. Cho Htway, a female MDR-TB patient staying at the TB village explained, “Health care providers arrange and help us. They encourage and support us. They provide food and accommodation, free treatment and love for us”. Patients expressed strong family-like relationships with the care providers in the TB villages and explained that staff helped them adhere to treatment by offering psychological encouragement. In both residential and supportive programs, migrants and refugees play a strong role in care provision serving as medics and community health volunteers.

An added bonus of residential treatment programs, beyond treatment adherence, is that family members who accompany the patient to the clinic are also screened. A female TB clinic doctor explained that screening accompanying family members has assisted in early detection of TB among contact children which can help to prevent the negative health consequences associated with late diagnosis, specifically TB meningitis and bone TB. She described that late presentation of TB in children is common in this population.

It is very severe, if they come in with late presentation. Even though we can combat the TB but we can’t solve the problem associated the neurological consequences (related to TB meningitis). And sometimes the destruction from spinal TB is really bad. They have to end up as a paraplegic or something like that.

Beyond residential programs, organizations have developed supportive programs to help ensure treatment adherence for migrant patients who were receiving treatment at the MSH. WVT organized direct observed treatment (DOT) therapy program to provide migrants with their daily TB medication at home in migrant communities surrounding Mae Sot. SMRU also had a project with MSH to visit TB patients at their residences. The funding for both projects was scheduled to end shortly after our team collected data for this project.

### Opportunity: Inter-organizational collaboration

Upon examining the key informant data we found that organizations that provide TB treatment to migrants and refugees in Tak province are actively collaborating regarding patient care and overall TB control among migrant and refugee populations. These collaborations include patient referrals and the provision of supportive services. Wiya Htoo, a male migrant health worker who also became a MDR-TB patient described the inter-organizational collaboration that eased his access to care,

After I realized I had TB, the staff from my clinic made a call to the TB doctor here (at the TB residential treatment village) and then she came to pick me up from the clinic and brought me here. Because of the easy communication I didn’t have to wait long to get treatment.

Patients in the refugee camp who have MDR-TB are also referred to SMRU for treatment. Treatment providers were also collaborating with WVT, which provided supportive services such as transportation and accommodation during treatment. Maya, a female migrant health volunteer expressed how the collaboration between WVT and the local government hospital benefited patients, “If we have to go to the clinic or the hospital, World Vision will provide free treatment for us, so we don’t have to worry about transportation fees and legal status”. World Vision Thailand established migrant health posts run by volunteers who can record symptoms, refer potential TB cases to doctors, collect sputum and send it to the lab as well as provide transportation during treatment and DOTs.

In terms of TB control among migrant and refugee populations, organizations that provide TB treatment or supportive care and the Tak PHO belong to the TTBI. The TTBI provides a forum for organizations to share data and to develop shared strategies to treat patients and reduce number of new TB cases in the border region. As a network the TTBI also applied for and obtained funding from the European Union and the United Kingdom Department for International Development, which was then distributed to members to pay for TB treatment programs run by member organizations.

## Discussion

Surveillance gaps in Tak province contribute to the invisibility of migrant TB cases. SMRU is the primary provider of TB care for migrants who have TB, TB/HIV, and MDR-TB in Tak province. By not integrating SMRU’s data into the provincial statistics, we anticipate that the burden of TB in migrant populations is under-reported. Under reporting of TB cases hampers TB control as it is difficult to identify trends and to evaluate efforts to reduce TB in migrant populations without an appropriate estimation of the actual burden. This work builds upon previous epidemiological studies, which suggest that the burden of TB is higher among migrant populations and thus an approximation of national prevalence figures from the provincial level is insufficient [9, 10, 12].

From a health services perspective, TB treatment provision for migrants in Tak province is tenuous. At the time of our research migrants could access TB treatment from government hospitals due to donor funding. However, treatment for TB/HIV and MDR-TB, which requires more complex care, was limited to one provider. Our finding that treatment provision is closely linked to donor funding has important implications as a gap in funding could eliminate treatment options for migrants with TB/HIV and MDR-TB. The treatment availability gap for migrants would subsequently also negatively impact TB control. Research participants identified a gap in TB care provision for migrant patients in Tak province when MSF left Thailand in 2011, however our team did not find documentation that describes the impact of this gap.

The findings on the opportunities for TB control in Tak province illustrate that the foundations for further interventions are sufficiently strong. First, organizations are already collaborating by sharing data, meeting to discuss TB control and referring patients. Second, NGOs are providing migrants with comprehensive medical treatment and supportive services, which are effective but also resource intensive. Organizations have developed a model of care where healthcare is provided in languages patients understand by Myanmar doctors, migrant and refugee medics and community health volunteers. Integrated HIV counseling, testing and treatment within these TB programs also provides an opportunity to enhance control over both diseases in the border region. Residential TB care plays an important role in the border region. Despite the disadvantages of residential treatment, specifically temporary loss of livelihood and separation from family, we found that given the mobile nature of patients, residential treatment in this context provides patients with the opportunity to receive and adhere to care. Residential treatment is especially beneficial for migrants who do not have a residence where home treatment could be provided. Third, targeted active case findings have the potential to enhance early case detection among migrants. Additionally, the use of the GeneXpert test, which can provide results within 2 hours, may lead to improved TB and MDR-TB diagnosis and treatment initiation in a mobile population. The increased sensitivity of GeneXpert over smear microscopy, can support the TB diagnosis of some smear negative patients which were missed by conventional microscopy [17,18]. On the Thailand-Myanmar border, 12% of GeneXpert positive cases at SMRU are smear negative. GeneXpert’s increased sensitivity over smear microscopy, may further decrease loss to follow up by avoiding additional diagnostic procedures required for clinical diagnosis of TB such as chest x-ray and antibiotic trial, which take an additional 7-10 days to conclude diagnosis. Moreover, in rapidly identifying multi-drug resistant TB, GeneXpert contributes to the early identification and treatment of MDR TB without needing to wait 4-8 weeks for a conventional culture and drug sensitivity result. In this research context, where migrants are highly mobile and are not easily reachable for follow up, we anticipate that reduced timeframes between TB testing and results can improve the likelihood that individuals will receive their results and subsequently begin treatment. We note that this observation is specific to a mobile population. In comparison, the XTEND study, a randomized control trial control trial comparing Xpert and sputum microscopy initial testing did not find a significant difference in loss to follow up between groups, however migrants were not included in the study as participants needed to reside in the clinic area without plans to relocate for 8 months [17]. On the Thailand-Myanmar border, where migrant populations are disproportionately burdened with MDR-TB, GeneXpert also provides clinicians with the opportunity to test for TB and begin treatment while waiting for the drug susceptibility result from growing and testing a TB culture [10].

The Thailand-Myanmar border in Tak province is a region that is undergoing a transition from a small border area to a large economic hub. With the rise of the ASEAN community has come promises of further economic integration between Myanmar and Thailand. An area close to the Thai border city of Mae Sot has been designated as a special economic zone and we anticipate the zone’s new factories will increase the number of migrants coming across the border to seek work in the formal and informal sectors [18]. As healthcare in Thailand is known regionally for its high quality, it is likely that in the future people will continue to cross the border to access healthcare in Tak province [10]. In addition, if the refugee camps close, residents who lived there may decide to continue living in Thailand and would then become part of the larger migrant population.

As migration to Tak province will likely increase, we suggest that Tak PHO explore adopting a reporting system which integrates all migrant cases regardless of their distinction as cross-border migrants who come across the border to access healthcare or migrant workers who are working and living in Thailand. The definition of migrant is fluid and we acknowledge that cross-border migrants may change their migration status to live in Thailand and migrant workers may become cross-border migrants. Furthermore, unlike refugees who are largely confined to the provinces’ refugee camps, migrants are not living separately from the majority Thai population. Enumerating the burden of TB in migrant populations will make it easier to plan further interventions to address it. Our findings support the suggestion made by previous research that public health officials from Thailand and Myanmar continue to work together to strengthen data sharing on TB cases between the two countries [11].

All stakeholders should be encouraged to continue to engage in collaborative planning around TB, TB/HIV, and MDR-TB treatment provision for migrant populations. It could be useful to explore alternative providers for MDR-TB and TB/HIV treatment provision for migrants as well as transition planning if the current single provider can no longer provide the service. MDR-TB is a serious threat to public health and is long, difficult and expensive to treat. Use of the World Health Organization newly validated short course regimen for MDR-TB treatment could assist with TB control along the Thailand-Myanmar border [19]. The short course regimen is more cost effective than conventional treatment and reduces the time for treatment from 18–24 to 9–12 months, which based on a fixed drug supply could increase treatment allocation as more patients could receive treatment given the reduction in cost and duration per person. The short course regimen could also decrease loss to follow up among migrant populations. Together, increased treatment allocation and reduced loss to follow up could potentially lower TB transmission and lessen the emergence of further drug resistant strains in this community. A modeling study in Uzbekistan suggested that of the short course regimen may decrease transmission of drug resistant TB [20]. In Thailand, further discussions may be necessary to examine how MDR-TB cases in migrant populations should be funded. The TTBI could work to address funding sustainability by investigating the possibility that the Thai government’s Compulsory Migrant Health Insurance Scheme, which provides primary healthcare access, could be expanded to include MDR-TB treatment for migrants, although additional subsidies might be required to make the scheme affordable for migrants. Another possibility is for Myanmar to help fund migrants’ TB treatment in Thailand through a direct fund transfer to treatment providers or through the development of a health insurance scheme for Myanmar citizens working abroad which is similar to what has been established in the Philippines [21]. Our findings support the suggestion that bi-national referral mechanisms be further developed, to allow patients to transfer between the two countries while reducing the likelihood that they will be lost to follow up [11]. This cross-border referral mechanism in addition to proper counseling may reduce concerns about drug resistance among treatment providers.

Internationally challenges with cross-border TB control have been identified along the Cameroon-Equatorial Guinea border, along the European Union’s (EU) eastern border and between EU countries [1–4]. The cross-border work on TB control along the Thailand-Myanmar border is nascent, particularly in comparison with Europe where World Health Organization experts have developed a consensus statement on a minimum cross border TB control and care. By providing an overview of challenges and opportunities for TB control in a middle income country border region, we anticipate that our results will contribute to ongoing efforts to enhance surveillance and treatment provision [1]. Health care providers and public health officials working in regions with a porous international border where availability, quality and cost of TB treatment varies significantly between nations may find this article useful as a comparator to their own experience. In analyzing their own situation, organizations may wish to consider whether all TB cases should be enumerated regardless of legal status, TB treatment sustainability, and what types of opportunities exist to further enhance TB control in their region.

### Limitations

The socio-economic situation along the Thailand-Myanmar border is rapidly changing and one limitation of this work is that the majority of our data was collected over a single 3-month period in 2014. Furthermore, due to logistical challenges we were only able to include key informants from two of the five government hospitals. As these data are qualitative we cannot generalize the results to all migrant and refugee TB patients and health care providers along the Thailand-Myanmar border. Given the resources available for this study, we only conducted data collection on the Thailand side of the border, and as such this study does not contribute to the literature regarding TB control on the Myanmar side of the border. However, this research provides some illustrative examples of the challenges and opportunities for TB prevention and care which may be of relevance to health care providers and public health officials who are working in similar international border contexts.

## Conclusion

Along the Thailand-Myanmar border in Tak province, Thailand, migrant and refugee populations are disproportionately burdened by TB. In investigating possibilities for enhanced TB control in these populations we identified surveillance, treatment, and potential funding gaps as well as opportunities in the areas of improved diagnostics, comprehensive medical and supportive care, and inter-organizational collaboration. We recognize that data sharing between countries, cross border referrals and a new MDR-TB short course regimen have the potential to positively contribute to TB control in this border region. Our findings suggest the need to enhance the surveillance system to include all migrant TB patients who seek treatment in Tak province and support efforts by stakeholders on both sides of the border to continue to share data and engage in collaborative planning on TB, TB/HIV, and MDR-TB treatment provision for migrant populations.

While the results of this study are specific to the Thailand-Myanmar border, the identified gaps and opportunities for TB control among migrant populations may be useful for other international border regions where the availability, quality, and cost of TB treatment varies significantly between nations.

## Abbreviations

AEC: ASEAN Economic Community
ASEAN: Association of Southeast Asian Nations
DOT: Directly observed treatment
FGD: Focus group discussion
IOM: International Organization for Migration
KI: Key informant
MDR-TB: Multidrug-resistant tuberculosis
MSF: Médecins Sans Frontières
MSH: Mae Sot Hospital
NGO: Non-governmental organization
PU-AMI: Première Urgence-Aide Médicale Internationale
SMRU: Shoklo Malaria Research Unit
Tak PHO: Tak Provincial Public Health Office
TB: Tuberculosis
TB/HIV: Tuberculosis and Human Immunodeficiency Virus Co-infection
TTBI: Tak Tuberculosis Initiative
WVT: World Vision Thailand
