# Erratum to: Treating the invisible: Gaps and opportunities for enhanced TB control along the Thailand-Myanmar border

**DOI:** 10.1186/s12913-017-2113-7

**Published:** 2017-03-16

**Authors:** Naomi Tschirhart, Sein Sein Thi, Lei Lei Swe, Francois Nosten, Angel M. Foster

**Affiliations:** 1 Faculty of Health Sciences, University of Ottawa, 1 Stewart Street, Ottawa, K1N 6N5 ON Canada; 2 Shoklo Malaria Research Unit, Mahidol-Oxford Tropical Medicine Research Unit, Faculty of Tropical Medicine, Mahidol University, PO Box 46, Mae Sot, Tak 63110 Thailand; 3 Centre for Tropical Medicine and Global Health, Nuffield Department of Clinical Medicine, University of Oxford, Churchill Hospital, Oxford, UK

## Erratum

After publication of the original article [1] it was brought to our attention that some of the authors’ final corrections were not incorporated in the online version of the paper.

1. Two references added by the author were not included in the reference list, resulting to several incorrect in-text citations. The omitted references are:
  19. Churchyard GJ, Stevens WS, Mametja LD, McCarthy KM, Chihota V, Nicol MP, et al. Xpert MTB/RIF versus sputum microscopy as the initial diagnostic test for tuberculosis: a cluster-randomised trial embedded in South African roll-out of Xpert MTB/RIF. Lancet Glob Heal. 2015; 3(8):e450–e457.
  20. Gray D. Development in Southeast Asia: From backwater to boomtown – Thailand’s Mae Sot. In: Nikkei Asian Review. http://asia.nikkei.com/Politics-Economy/Economy/From-backwater-to-boomtown-Thailand-s-Mae-Sot AQ15 2015. Accessed 1 Apr 2016.

The above references have been included in the reference list and both the subsequent references and in-text citations have been renumbered accordingly in the original article.

2. During the production process of the final article the phrase “HIV for this population. Since” was erroneously inserted into the article text multiple times where it should read "of TB". Therefore, the following sentences have been revised as outlined below
  - In the 'Background' the sentence "From a surveillance perspective, data collection remains a barrier to effective TB control as public health departments struggle to document the number ***HIV treatment for this population*** *.* ***Since*** cases among mobile patients in their jurisdiction as well as to share and collaborate with their counterparts on the other side of the national border" has been revised to read: "From a surveillance perspective, data collection remains a barrier to effective TB control as public health departments struggle to document the number of TB cases among mobile patients in their jurisdiction as well as to share and collaborate with their counterparts on the other side of the national border".
  b. In the 'Background' the sentence "A 2007 study also found that the majority ***HIV treatment for this population*** *.* ***Since*** cases (65%) were among non-Thais in Tak province and estimated the prevalence to be 109 per 100,000 for Thai citizens, 340 per 100,000 for Myanmar refugees, and 150 per 100,000 for Myanmar migrants [10]" has been revised to read: "A 2007 study also found that the majority of TB cases (65%) were among non-Thais in Tak province and estimated the prevalence to be 109 per 100,000 for Thai citizens, 340 per 100,000 for Myanmar refugees, and 150 per 100,000 for Myanmar migrants [10]".
  c. In the 'Background' the sentence "We use the term “TB control” throughout to indicate TB care and prevention efforts and acknowledge that TB control is not solely dependent on public health specialists but also relies to the resources **HIV treatment for this population. Since** patients and their families [5]" has been revised to read: "We use the term “TB control” throughout to indicate TB care and prevention efforts and acknowledge that TB control is not solely dependent on public health specialists but also relies to the resources of TB patients and their families [5]".

d. In the 'Data collection' section of 'Methods' the sentence "To be eligible to participate, patients needed to have a confirmed case **HIV treatment for this population. Since**, MDR-TB or TB/HIV" has been revised to read: "To be eligible to participate, patients needed to have a confirmed case of TB, MDR-TB or TB/HIV".

The above corrections have been incorporated in the original version of this article.
